# Mississippi Valley Dental Association

**Published:** 1895-05

**Authors:** 


### EDITORIAL.

### Mississippi Valley Dental Association.

The semi-annual meeting of this body was held in the Odd Fellows' Temple, Seventh and Elm Streets, April 17th and 18th; about one hundred persons being present. A very interesting programme of subjects had been prepared by the Executive Committee. The persons to whom subjects had been assigned accomplished the work expected of them. Every subject on the programme was presented by paper and fully discussed. The papers were of a high order and the discussions were much above the average. This may be attributed to the fact that the various subjects of the papers were assigned to individuals from two to three to each paper for disscussion. The papers were in the hands of those who were to discuss them, sometime before the meeting, so that they came fully prepared for the work before them, and right well was this duty accomplished. We have never, in any dental association, heard better, if as good, so systematic and well arranged discussion as on this occasion. For the past three or four years the members seemed somewhat to have lost interest in this society, but through the persistent efforts of the Executive Commitee, this meeting was made an eminent success, though last year there was a strong sentiment in favor of dissolving the society. The present meeting has been better in every respect than has been held by the same body for the last twenty-five years, and indeed it is a question whether, if ever, in the history of the society, a better meeting has been held. An enthusiasm was engendered that is prophetic of its success for years to come.

There were present quite a number of the older members from distant points.

The election of officers for the ensuing year were as follows:

President, Dr. William V. B. Ames, Chicago; First Vice-President, Dr. J. E. Cravens, Indianapolis; Second Vice-President, Dr. H. C. Matlack, Cincinnati; Treasurer, Dr. F. A. Hunter, Cincinnati; Recording Secretary, Dr. H. T. Smith, Cincinnati.

The Executive Committee are as follows:

Drs. J. R. Callahan, M. H. Fletcher, of Cincinnati; L. E. Custer, of Dayton, Ohio.

The Executive Committee consist of the same persons as last year.

The following were elected delegates to The American Dental Association, which meets at Asbury Park, New Jersey, on the first Tuesday of August next: J. E. Cravens, of Indianapolis; A. P. Bollinger, of Dayton, Ohio; H. C. Matlack, of Cincinnati; W. S. Locke, Cincinnati; W. B. Chambers, of Newark, Ohio;

W. D. Phillips, of New Vienna, Ohio; and Frank Sage, Sarah S. Harris, Jessie Dillon and Viola Swift, of Cincinnati.

The meeting continued two days, holding only day sessions, and never, perhaps, in any similar body, was more good work crowded into so short a space of time.

The papers and discussions will be published in full in subsequent numbers of "THE REGISTER."

